# *Notes from the Field:* Use of Electronic Cigarettes and Any Tobacco Product Among Middle and High School Students — United States, 2011–2018

**DOI:** 10.15585/mmwr.mm6745a5

**Published:** 2018-11-16

**Authors:** Karen A. Cullen, Bridget K. Ambrose, Andrea S. Gentzke, Benjamin J. Apelberg, Ahmed Jamal, Brian A. King

**Affiliations:** ^1^Center for Tobacco Products, Food and Drug Administration, Silver Spring, Maryland; ^2^Office on Smoking and Health, National Center for Chronic Disease Prevention and Health Promotion, CDC.

Electronic cigarettes (e-cigarettes) are battery-powered devices that provide nicotine and other additives to the user in the form of an aerosol (*1*). E-cigarettes entered the U.S. marketplace in 2007 (*1*), and by 2014, e-cigarettes were the most commonly used tobacco product among U.S. youths (*2*). Data from the 2011–2018 National Youth Tobacco Survey (NYTS), a cross-sectional, voluntary, school-based, self-administered, pencil-and-paper survey of U.S. middle and high school students, were analyzed to determine the prevalence of current use (≥1 day in past 30 days) of e-cigarettes,* current use of any tobacco product,^†^ frequency of (number of days during the preceding 30 days) e-cigarette use, and current use (any time during preceding 30 days) of any flavored e-cigarettes among U.S. middle school (grades 6–8) and high school (grades 9–12) students. Logistic regression (2011–2018) and t-tests (2017–2018) were performed to determine statistically significant differences (p<0.05).

Among high school students, current e-cigarette use increased from 1.5% (220,000 students) in 2011 to 20.8% (3.05 million students) in 2018 (p<0.001) (Figure). During 2017–2018, current e-cigarette use increased by 78% (from 11.7% to 20.8%, p<0.001). The proportion of current e-cigarette users who reported use on ≥20 of the past 30 days increased from 20.0% in 2017 to 27.7% in 2018 (p = 0.008). Among high school students, during 2017–2018, current use of any flavored e-cigarettes increased among current e-cigarette users (from 60.9% to 67.8%, p = 0.02); current use of menthol- or mint-flavored e-cigarettes increased among all current e-cigarette users (from 42.3% to 51.2%, p = 0.04) and current exclusive e-cigarette users (from 21.4% to 38.1%, p = 0.002).

**FIGURE:**
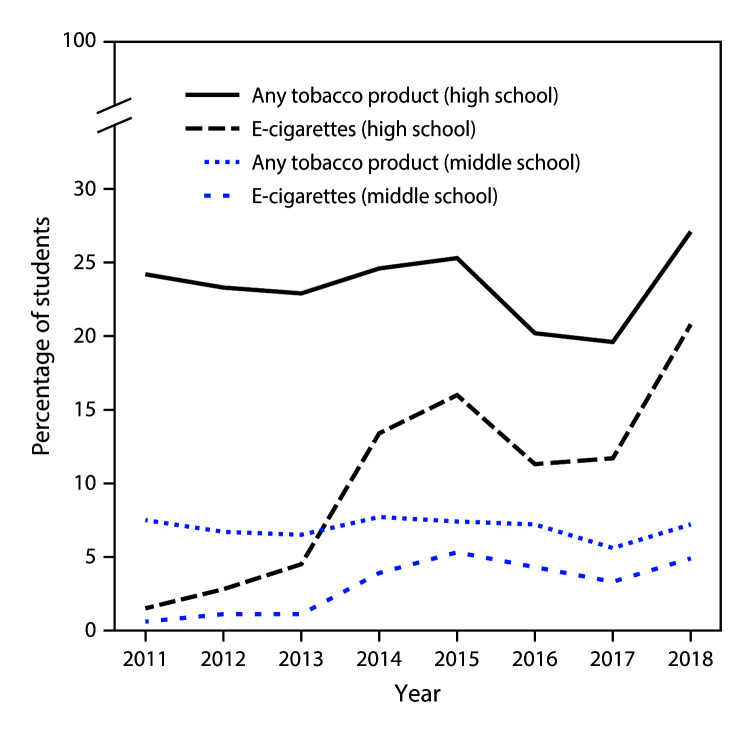
Percentage of middle and high school students who currently use e-cigarettes* and any tobacco product^†^ — National Youth Tobacco Survey, United States, 2011–2018 * Current e-cigarette use was assessed by responses to these questions during the indicated survey years: “In the past 30 days, which of the following products have you used on at least one day?” and the response option, “Electronic cigarettes or e-cigarettes such as Ruyan or NJOY” (2011–2013); “During the past 30 days, on how many days did you use e-cigarettes such as Blu, 21st Century Smoke, or NJOY?” (2014); “During the past 30 days, on how many days did you use electronic cigarettes or e-cigarettes?” (2015); and “During the past 30 days, on how many days did you use e-cigarettes?” (2016–2018). During 2015–2018, e-cigarette questions were preceded by an introductory paragraph defining the product. ^†^ Any tobacco product was defined as use of one or more of the following tobacco products on ≥1 day in the past 30 days: cigarettes, cigars (defined as cigars, cigarillos, or little cigars), smokeless tobacco (defined as chewing tobacco, snuff, or dip), e-cigarettes, hookahs, tobacco pipes, snus, dissolvable tobacco, and bidis.

Among middle school students, current e-cigarette use increased from 0.6% in 2011 (60,000 students) to 4.9% (570,000 students) in 2018 (p<0.001) (Figure). During 2017–2018, current e-cigarette use increased by 48% (from 3.3% to 4.9%, p = 0.001); the proportion of current e-cigarette users who reported use on ≥20 days of the past 30 days did not significantly change (from 12.9% to 16.2%, p = 0.26).

Current use of any tobacco product among high school students was 24.2% (3.69 million students) in 2011 and 27.1% (4.04 million students) in 2018 (p>0.05) (Figure). Current use of any tobacco product among middle school students was 7.5% (870,000 students) in 2011 and 7.2% (840,000 students) in 2018 (p>0.05). During 2017–2018, overall tobacco product use increased by 38% among high school students (from 19.6% to 27.1%, p<0.001) and by 29% among middle school students (from 5.6% to 7.2%, p = 0.008).

Current e-cigarette use increased considerably among U.S. middle and high school students during 2017–2018, reversing a decline observed in recent years and increasing overall tobacco product use (*3*). Moreover, during 2017–2018, frequent e-cigarette use increased among high school students. Although e-cigarettes have the potential to benefit adult smokers if used as a complete substitute for combustible tobacco smoking, the use of any form of tobacco product among youths, including e-cigarettes, is unsafe (*1*). The Surgeon General has concluded that e-cigarette use among youths and young adults is of public health concern; exposure to nicotine during adolescence can cause addiction and can harm the developing adolescent brain (*1*). 

The rise in e-cigarette use during 2017–2018 is likely because of the recent popularity of e-cigarettes shaped like a USB flash drive, such as JUUL; these products can be used discreetly, have a high nicotine content, and come in flavors that appeal to youths (*4*). In September 2018, the Food and Drug Administration (FDA) issued more than 1,300 warning letters and civil money penalty fines to retailers who illegally sold e-cigarette products to minors, the majority of which were blu, JUUL, Logic, MarkTen XL, and Vuse; this was the largest coordinated enforcement effort in FDA’s history (*5*). Sustained implementation of proven population-based strategies, in coordination with the regulation of tobacco products by FDA, is key to reducing all forms of tobacco product use and initiation, including e-cigarettes, among U.S. youths (*1*).

## Footnotes

* Current e-cigarette use was defined as a response greater than “0 days” to the question, “During the past 30 days, on how many days did you use e-cigarettes?” The e-cigarette questions were preceded by the following information: “The next 11 questions are about electronic cigarettes or e-cigarettes. E-cigarettes are battery powered devices that usually contain a nicotine-based liquid that is vaporized and inhaled. You may know them as e-cigs, vape-pens, hookah-pens, e-hookahs, e-cigars, e-pipes, personal vaporizers, or mods. Some brand examples include NJOY, blu, Vuse, MarkTen, Logic, Vapin Plus, eGo, and Halo.”

† Any tobacco product use was defined as use of one or more of the following tobacco products on ≥1 day in the past 30 days: cigarettes, cigars (defined as cigars, cigarillos, or little cigars), smokeless tobacco (defined as chewing tobacco, snuff, or dip), e-cigarettes, hookahs, tobacco pipes, snus, dissolvable tobacco, and bidis.
